# One Health assessment of persistent organic chemicals and PFAS for consumption of restored anadromous fish

**DOI:** 10.1038/s41370-023-00620-3

**Published:** 2023-12-15

**Authors:** Lisa Jo Melnyk, James M. Lazorchak, Daniel H. Kusnierz, Gary D. Perlman, John Lin, Raghuraman Venkatapathy, Devi Sundaravadivelu, Jonathan Thorn, James Durant, Katherine Pugh, Michael A. Stover

**Affiliations:** 1 U.S. Environmental Protection Agency (EPA), Office of Research and Development, Cincinnati, OH USA; 2 Penobscot Indian Nation, Department of Natural Resources, Indian Island, ME USA; 3 Senior Environmental Employee Grantee, EPA, Region 1, Boston, MA USA; 4 Pegasus Technical Services, Inc., Cincinnati, OH USA; 5 Battelle, Norwell, MA USA; 6 Agency for Toxic Substances and Disease Registry, Office of Community Health and Hazard Assessment, Atlanta, GA USA; 7 EPA, Region 4, Atlanta, GA USA; 8 EPA, Region 1, Boston, MA USA

**Keywords:** Anadromous fish, Exposure, PFAS, Persistent organic chemicals, One health approach

## Abstract

**Background:**

Restoration efforts have led to the return of anadromous fish, potential source of food for the Penobscot Indian Nation, to the previously dammed Penobscot River, Maine.

**Objective:**

U.S. Environmental Protection Agency (EPA), Penobscot Indian Nation’s Department of Natural Resources (PINDNR), and Agency for Toxic Substances and Disease Registry (ATSDR), measured contaminants in six species of anadromous fish. Fish tissue concentrations were then used, along with exposure parameters, to evaluate potential human and aquatic-dependent wildlife risk.

**Methods:**

PINDNR collected, filleted, froze, and shipped fish for analysis of polychlorinated biphenyls (PCBs), polybrominated diphenyl ethers (PBDEs), dioxins/furans, and per- and polyfluoroalkyl substances (PFAS). Contaminant levels were compared to reference doses (where possible) and wildlife values (WVs).

**Results:**

Chemical concentrations ranged from 6.37 nanogram per gram (ng/g) wet weight (ww) in American Shad roe to 100 ng/g ww in Striped Bass for total PCBs; 0.851 ng/g ww in American Shad roe to 5.92 ng/g ww in large Rainbow Smelt for total PBDEs; and 0.037 ng/g ww in American Shad roe to 0.221 ng/g ww in Striped Bass for total dioxin/furans. PFAS concentrations ranged between 0.38 ng/g ww of PFBA in Alewife to 7.86 ng/g ww of PFUnA in Sea Lamprey. Dioxin/furans and PFOS levels indicated that there are potential human health risks. The WV for mink for total PCBs (72 ng/g) was exceeded in Striped Bass and the WV for Kestrel for PBDEs (8.7 ng/g) was exceeded in large Rainbow Smelt. Mammalian wildlife consuming Blueback Herring, Striped Bass, and Sea Lamprey may be at risk based on PFOS WVs from Canada.

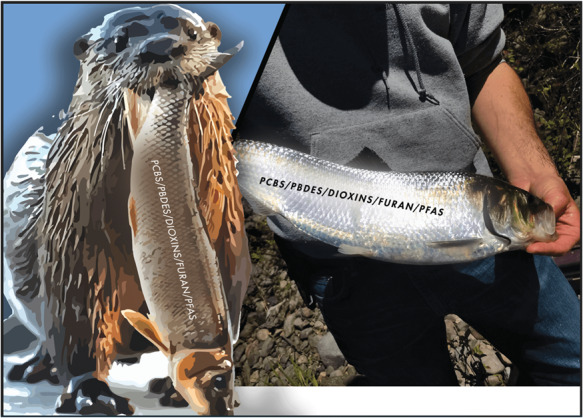

**Impact:**

- Anadromous fish returning to the Penobscot River potentially could represent the restoration of a major component of tribal traditional diet. However, information about contaminant levels in these fish is needed to guide the tribe about consumption safety. Analysis of select species of fish and risk calculations demonstrated the need for a protective approach to consumption for both humans and wildlife. This project demonstrates that wildlife can also be impacted by contamination of fish and their risks can be as great or greater than those of humans. A One Health approach addresses this discrepancy and will lead to a healthier ecosystem.

## Introduction

Fish consumption advisories developed by states and tribes are commonly used to ensure adequate human health protections against Persistent Organic Chemicals (POC) that have been measured in fish [1, 2]. Sources of POC contamination in fish tissue are varied and dependent on where fish are, uses in the area (such as industrial), and environmental factors. Polychlorinated biphenyls (PCBs), polybrominated diphenyl ethers (PBDEs), dioxin/furans, and per- and polyfluoroalkyl substances (PFAS) are some of the most widely studied contaminants measured in fish. Several studies have evaluated these organic contaminants in fish in the Great Lakes region of the United States and Canada [3–10]. Other studies have focused on fish contamination in rivers [11], harbors [12, 13], or as part of a dietary intake study [14, 15].

Initially focus was placed on perfluorooctanesulfonic acid (PFOS) and perfluorooctanoic acid (PFOA) but as industry increased production of other PFAS, more substances have become the focus of studies. An association between elevations of several PFAS compounds in human serum and intake of certain fish and shellfish was reported based on data collected as part of the U.S. Centers for Disease Control and Prevention (CDC’s) National Health and Nutrition Examination Survey (NHANES) [16]. The European Food Safety Authority [14], having determined that diet is a main source of exposure to PFAS in Europe, identified consumption of fish as one of the primary dietary sources.

Human exposure to persistent contaminants is of great concern, but the ecological impact is equally as important to understand. Fish, a major source of exposure to these compounds, are consumed by both humans and wildlife. It is imperative that each are considered in assessing the impact of contamination in fish resulting in a One Health approach [17].

The U.S. Environmental Protection Agency (EPA), in collaboration with the Penobscot Indian Nation’s Department of Natural Resources (PINDNR) and the Agency for Toxic Substances and Disease Registry (ATSDR), examined the potential risk of human and wildlife consumption of six species of anadromous fish (fish that live as adults in salt water, and spawn in fresh water). The purpose of the study was to measure POC including PFAS levels in anadromous fish collected from the Penobscot River, Maine during two consecutive spawning seasons (2017 and 2018). Potential impacts were previously evaluated for mercury in anadromous fish collected from the Penobscot River [18]. These results will provide information that can be used by states and tribes in determining safe consumption amounts of each fish type for both humans and wildlife.

## Methods

### Fish preparation and analysis

**Fig. 1:**
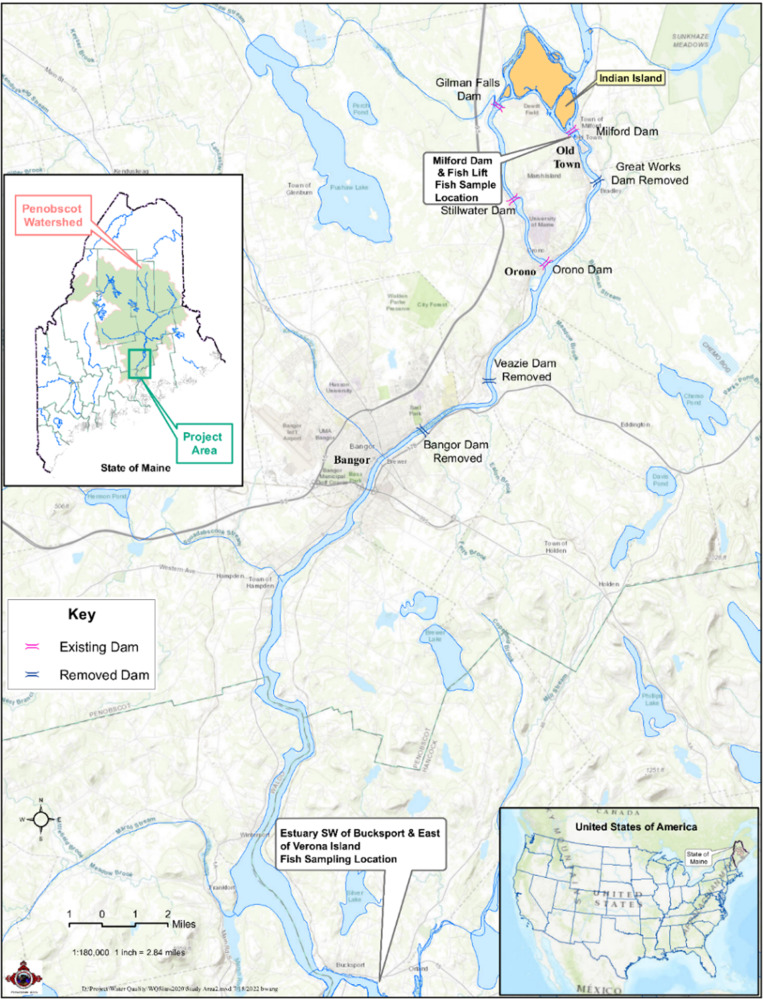
Map of the study area with sampling sites for fish collection. Sampling site on the Penobscot River, Maine. Inset 1—the United States of America. Inset 2—State of Maine showing the project area and Penobscot Watershed.

Anadromous fish species were collected during their upstream spawning migration in the Penobscot River in Maine. Figure 1 depicts the sampling location for this study. The fish species collected included Alewife (*Alosa pseudoharengus*), American Shad (*Alosa sapidissima*), Blueback Herring (*Alosa aestivalis*), Rainbow Smelt (*Osmerus mordax*), Striped Bass (*Morone saxatilis*), and Sea Lamprey (*Petromyzon marinus*). Fish size was chosen to be consistent with EPA National Survey methods [19]. Roe samples were also collected from the American Shad. Briefly, the fish were collected from the Penobscot River and processed at the PINDNR laboratory. The PINDNR followed a procedure to create composites of each species (Table 1); details of the fish collection and processing are provided in Melnyk et al., 2021 (ref. 18) with an additional processing step that includes shipping leftover composite homogenates of fish samples to a separate laboratory that has capabilities to analyze PFAS.

**Table 1:** Concentration with (95% confidence intervals) of persistent organic chemicals in anadromous fish collected from Penobscot river.

Species	Year	Composite samples	Number of fish per composite	Percent moisture	Percent lipids	Composite, nanogram/gram [wet weight] (3 replicates/composite averaged)		
						Total PCBs	Total PBDEs	Dioxin/Furans^a^
Alewife	2017	5	6	76.3	1.9	14.3 (11.6–17)	1.92 (1.44–2.39)	0.084 (0.066–0.103)
	2018	5	7	77.1	2.6	10.3 (9.23–11.4)*	1.74 (1.67–1.8)	0.091 (0.079–0.103)
American Shad Roe	2017	5	7	70.6	5.1	6.37 (4.37–8.37)	0.851 (0.66–1.04)	0.061 (0.049–0.074)
	2018	6	5	75.1	3.4	8.65 (7.48–9.82)	1.18 (0.838–1.51)	0.037 (0.033–0.041)**^,^^b^
American Shad Fillet	2017	5	3	75.1	7.8	24.2 (18.9–29.4)	2.73 (2.03–3.43)	0.078 (0.064–0.093)^a^
	2018	6	3	73.3	3.6	14.9 (13.4–16.3)**	2.44 (2.34–2.53)	0.105 (0.097–0.113)**
Blueback Herring	2017	5	5	76.2	3.4	17.2 (14.5–20)	2.16 (1.78–2.54)	0.062 (0.053–0.070)
	2018	5	6	76.5	2.8	19.2 (16.4–22.1)	2.31 (2.06–2.55)	0.065 (0.042–0.087)
Rainbow Smelt	2017	5	3	81.6	1.2	10.2 (3.57–16.9)	2.30 (1.54–3.07)	0.137 (0.096–0.177)
Rainbow Smelt, large	2018	3	10	79.8	1.6	18.6 (13–24.3)	5.92 (3.93–7.91)	0.125 (0.103–0.147)
Rainbow Smelt, small	2018	3	10	80.0	1.4	16.9 (15.8–18)	5.15 (4.41–5.88)	0.124 (0.111–0.136)
Striped Bass	2017	5	5	77.6	1.4	81.9 (45–119)	3.85 (3.26–4.43)	0.190 (0.074–0.306)
	2018	5	5	79.9	0.6	100 (29.2–171)	3.83 (3.02–4.63)	0.221 (0.186–0.256)
Sea Lamprey	2017	6	3	76.4	5.0	14.7 (8.86–20.6)	1.21 (0.883–1.55)	0.053 (0.035–0.072)
	2018	6	3	78.0	3.3	18.1 (4.61–31.5)	1.86 (1.26–2.45)	0.053 (0.038–0.067)

Survey based *t*-test *p < 0.05; **p < 0.01.

^a^Total sum of dioxins and furans. See Supplementary Table S3 for 2,3,7,8-TCDD TEQ.

^b^One replicate had rate of nondetectable levels too high for Kaplan–Meier estimation, so total calculated using ½ detection limit.

Tissue samples from the fillet or whole portions of the fish typically consumed by the population were analyzed for 32 PCB congeners, 27 PBDEs, and 17 dioxins/furans (see Supplementary Table S1). The contaminant list included all 12 PCB and 17 dioxin/furan compounds recommended by the World Health Organization (WHO) for risk assessment of dioxin-like compounds [20]. Seventy-five composite samples were shipped to Pegasus Technical Services, Inc (Cincinnati, OH) for analysis of PCBs, PBDEs, and dioxins/furans following established procedures for sample receiving, shipping, and processing. Upon receipt, the entire contents of the fish composites were homogenized using a stainless-steel blender (Waring, Torrington CT) and mixed to a fine paste of uniform color and texture. In 2017, 36 samples were processed; in 2018, 39 samples were processed. All samples were measured for moisture and lipid content. All analyses were performed in triplicate. Samples were preserved at −20 degrees Celsius, with a 40-day holding time for PCB extracts and an indefinite holding time for all other analyses.

For PFAS, 60 of the original 75 composite fish samples were shipped to Battelle (Norwell, MA). Not all of the initial samples could be shipped for PFAS analysis; Rainbow Smelt had insufficient amounts following PCBs, PBDEs and dioxins/furans analyses so none were shipped; Blueback Herring had reduced quantities, so only 3 composites from the 2017 batch were shipped; and one 2017 Striped Bass sample was not analyzed for PFAS. The samples were analyzed for 13 PFAS. The Supplemental Information contains the specific methods for measurements of moisture and lipid content, POC, and PFAS analyses. All analyses followed quality assurance and quality control processes as detailed in the Supplemental Information.

### Human health evaluation

The average concentration of POC contaminants in fish tissue was used to calculate an exposure dose (equation is provided in the Supplemental Information). The exposure dose was then compared to reference doses (RfDs) to obtain a non-cancer hazard quotient (HQ) that can be used to evaluate potential health implications. Specifically, a HQ < 1 indicates minimal risk when fish are consumed at the levels established for the tribal community for the specific POC from the Penobscot River [21].

The ingestion rate used for the community was 40 g/day (10 oz/week) for adult tribal members, which is the fish advisory ingestion rate from the Penobscot Indian Nation Guidelines for eating fish from Penobscot territory waters [22]. The exposure factor, which considers the frequency of exposure (e.g., days per year) is 1 for daily exposure (365 days/365 days). A body weight (BW) of 80 kg was used as recommended in EPA’s Exposure Factors Handbook (ref. 23). The RfDs for PCBs (Arochlor 1016 [24] or Arochlor 1254 [24]), PBDEs (BDE-99 [24]), and dioxin (2,3,7,8-tetrachlorodibenzo-p-dioxin (2,3,7,8-TCDD [24]), are 70, 20, 100, and 0.0007 ng/kg/day, respectively, as provided by EPA’s Integrated Risk Information System (IRIS) [24]. IRIS has published reference doses for PFAS, including but not limited to PFHxA (RfD, 5 × 10^−4 ^mg/kg-day) and PFBA (RfD, 1 × 10^−3 ^mg/kg-day) [24]. The EPA Office of Water (OW) has proposed a draft noncancer RfD of 1 × 10^−7 ^mg/kg-day [25] for PFOS, which was used in the human health evaluation.

Some POCs (PCBs and dioxin/furans) may increase the risk of developing cancer. IRIS lists an oral slope factor for one dioxin (HxCDD of 6.2 × 10^−3 ^mg/kg/day^−1^) [24], however, cancer risk was estimated using the oral cancer slope factor (1.3 × 10^5 ^mg/kg-day^−1^) as defined by the California Office of Environmental Health Hazard Assessment because it is inclusive of 2,3,7,8-TCDD and related compounds combined and represents the POCs of interest [26]. Cancer risk estimates are presented as the number of extra cancer cases in a group of similarly exposed people. For example, an estimated lifetime cancer risk might be 1 extra cancer case for every 10,000 people (1 × 10^–4^) who eat 40 grams of anadromous fish daily for 30 years for a 78-year life expectancy [27]. The estimated risk is not an actual number of cancer cases expected in a community and does not indicate an individual’s risk of developing cancer.

Toxic equivalents (TEQs) were used to express the numerous chemicals’ overall toxicity as a single value for chemicals in the same class with similar toxicological properties. TEQs were calculated for dioxin and dioxin-like compounds, including all 12 coplanar PCB’s [20, 27]. They were calculated to represent the overall toxicity of complex mixtures. In the case of dioxin, the toxicity of each individual congener was weighted against that of 2,3,7,8-TCDD, historically considered the most toxic member of these chemical classes [23, 28]. For these cancer risk calculations, collection year was not considered; therefore, all results were combined for each species and presented as one risk value. Values greater than 1 × 10^−4^ represent a public health concern for a potential increase in cancer risk [29]. The potential quantitative risk of cancer from PFAS exposure was not evaluated in this paper.

### Wildlife assessment

Wildlife also consume fish, resulting in potential exposure to POC and PFAS contaminants. Wildlife, however, consume the entire fish and not only the fillets, as humans do, therefore, the results of the composite fish samples were converted to an equivalent whole fish concentration to evaluate aquatic-dependent wildlife exposure [28]. Each chemical group had a unique conversion factor to evaluate the equivalent whole fish concentration; total PCB and total dioxin/furan results were multiplied by 1.83; total PBDEs results were multiplied by 1.5 [28]. The only PFAS with a conversion factor was PFOS, so PFOS results were multiplied by 2.13 [13]. The converted concentrations were compared to wildlife values (WVs) to determine if any potential risks to wildlife may be associated with exposure to POCs or PFOS.

Following the methods of Batt et al., 2017 (ref. 11), WVs were used to calculate potential risk. The WVs were 72 ng/g for total PCBs for Mink and 8.7 ng/g for total PBDEs for Kestrel [11]. The WV for dioxins/furans was 6.2 × 10^−5 ^ng/g [30], which was derived by multiplying the water quality standard (3.1 × 10^−9 ^µg/L) by the Bioconcentration Factor (15,000) or Bioaccumulation Factor (25,000), as stated in the reference, and taking the arithmetic mean. For dioxins, only 2,3,7,8-TCDD-was screened because of differing bioconcentration factors for the different congeners. For PFOS, the values established by Environment and Climate Change Canada were used where the Canadian WV for mammals is 4.6 ng/g and for birds is 8.2 ng/g [31].

### Statistical analysis

R version 4.1.2 [32] was used for data analysis and visualization. To assess multivariate differences, principal component analysis, biplots (See Statistical Analysis- biplots, Supplemental Information), and analysis of similarity after ranking the data using U-scores were used [33, 34]. For estimates of total PCBs, PBDE, and Dioxin/furan compounds, nondetectable concentrations were accounted for in the totals by using the Kaplan–Meier method to obtain a total estimate [35]. Because technical replicates were analyzed from each composite sample, the survey package version 4.1-1 [36] was used to perform summary statistics (mean, 95% confidence intervals using delta method) and to perform pairwise comparisons across size and year strata using survey design modified two-sided t-tests. The use of the survey methods is to account for clustering using the complex design with cluster set to composite sample identifier.

For PFAS compounds, EnvStats 2.50 [37] was used to estimate means and 95% confidence limits of the means and to assess goodness of fit. Kruskal–Wallis one way analysis of variance was used to compare for differences across multiple groups, while Wilcoxon rank sum tests were used to compare differences between groups.

## Results

### Levels of total persistent organic chemicals

To summarize the POC results, the averages of each measured compound were summed to present a total for PCBs, PBDEs, and dioxin/furans for each species. In 2018, the sizes of rainbow smelt varied more than the compositing criteria would allow, so separate averages for large and small smelt were created. Table 1 presents the results for the summary of concentrations for the POCs, with Supplementary Table S3 detailing the 2,3,7,8-TCDD toxicity equivalents (TEQs) for dioxin like compounds (including 12 coplanar PCBs). The ranges of chemical concentrations for both years combined were 6.37 ng/g ww in American Shad roe to 100 ng/g ww in Striped Bass for total PCBs; 0.851 ng/g ww in American Shad roe to 5.92 ng/g ww in large Rainbow Smelt for total PBDEs; and 0.037 ng/g ww in American Shad roe to 0.221 ng/g ww in Striped Bass for total dioxin/furans.

Generally, the PCB concentrations within the fish were greater than the PBDE concentrations, and the PBDE concentrations were greater than the dioxin/furans concentrations. The results are presented in ng/g wet weight, so the percent moisture (71–82%) is supplied to calculate dry weight values. The percent lipids are also included in Table 1 to allow for corrections, if needed. The amount of lipids in all fish species was below 8%.

Individual PCBs were detected in most of the samples for each species (97–100% for 2017 and 2018 sampling years combined) and detection rates for individual PBDEs in all the composite sample replicates were 62–89%. Dioxin/furans had the lowest concentrations and the lowest detection rates at 27–67% of the samples.

An Analysis of Similarity (ANOSIM) comparison of concentrations of contaminant levels in fish tissue between 2017 and 2018 found that PBDEs and dioxin/furans were significantly different (*p* < 0.05) between the two years, whereas PCBs were not different between years (*p* = 0.09). Biplots of the ranks of the concentrations show that the patterns of contamination for PCBs, PBDEs, and dioxin/furans differed by species (Supplementary Figs. S1–S3), (ANOSIM *p* < 0.05).

Total PCBs decreased from 2017 to 2018 in Alewife and American Shad Fillet. The biplots show the higher molecular weight PCBs decreased in Alewife and American Shad Filet from 2017 to 2018, while Striped Bass had the highest concentrations of PCBs compared to other species for both years (Supplementary Fig. S1). The multivariate pattern of PBDE results was not different by year. Biplots reveal that Striped Bass tended to have consistently higher PBDE concentrations with some Sea Lamprey, Rainbow Smelt and Blueback Herring samples also having higher concentrations (Supplementary Fig. S2). Total dioxin/furans were lower in 2018 for American Shad roe, and higher for American Shad Filet (Table 1). As shown on the biplot, Striped Bass tended to have higher polychlorinated difuran concentrations compared to other species. Rainbow Smelt results could not be compared between 2017 and 2018 because of differing fish lengths between years.

### Levels of per and polyfluoroalkyl substances (PFAS)

A reduced set of fish samples were analyzed for PFAS and the results are summarized in Table 2. Thirteen individual analytes were measured, but only 6 had detectable levels in any of the fish species. For this reason, a total PFAS concentration may not provide an accurate picture of the potential contamination within these fish species. The PFAS concentrations ranged between 0.38 ng/g ww of PFBA in Alewife to 7.86 ng/g ww of PFUnA in Sea Lamprey. The most detected PFAS was PFOS. Sea Lamprey contained more of a variety of PFAS than any other fish species (five out of the 6 detected). PFOS in American Shad roe increased in concentration from 2017 to 2018. Most of the species contained measurable levels of PFOS (except Alewife and American Shad Filet). PFDA, PFUnA, PFDoA, PFOSA and PFOS were found in Sea Lamprey, indicating Lamprey may be exposed to possible alternative sources of PFAS compared to other fish. ANOSIM indicated a variation of PFAS concentration by species (*p* < 0.05) and by year (ANOSIM *p* < 0.05). The Kruskal–Wallis Rank Sum Test indicated a significant difference between fish species (*p* < 0.01) for PFDA, PFUnA, PFDoA, PFOSA, and PFOS. Biplots of the ranks of the concentrations show that the patterns of contamination for PFAS were different by species (Supplementary Fig. S4).

**Table 2:** Concentration with (95% confidence intervals) of PFAS in anadromous fish collected from Penobscot river, nanogram per gram [wet weight].

Species	Year	Composite Samples	PFBA	PFDA	PFUnA	PFDoA	PFOSA	PFOS
Alewife	2017	5	<0.38^a^	<DL	<DL	<DL	<DL	<DL
	2018	5	<DL^b^	<DL	<DL	<DL	<DL	<DL
American Shad Roe	2017	5	1.11	<DL	<DL	<DL	<DL	2.65 (1.17–3.88)
	2018	6	<DL	<DL	<DL	<DL	<DL	5.38 (4.26–6.49)**
American Shad Fillet	2017	5	<DL	<DL	<DL	<DL	<DL	<DL
	2018	5	<DL	<DL	<DL	<DL	<DL	<DL
Blueback Herring	2017	3	<DL	<DL	<DL	<DL	<DL	4.18 (1.42–6.95)
	2018	5	<DL	<DL	<DL	<DL	<DL	<DL*
Striped Bass	2017	4	<DL	<DL	<DL	<DL	1.57 (1.01–2.14)	3.21 (1.14–5.29)
	2018	5	<DL	<DL	<DL	<DL	1.11 (0.839–1.34)	2.26 (1.46–3.05)
Sea Lamprey	2017	6	<DL	2.74 (0.531–4.67)	7.86 (2.25–13.5)	1.59 (0–2.70)^c^	5.92 (3.20–8.63)	6.59 (0–13.8)^c^
	2018	6	<DL	1.52 (0.914–2.06)	3.75 (2.22–5.29)	1.01 (0.25–1.36)	5.07 (2.23–7.91)	5.84 (2.25–9.44)

*p < 0.05; **p < 0.01.

^a^Only 1 sample had detectable PFBA (1.11 ng/g). Confidence interval could not be estimated.

^b^Below detection limit for PFBA of 0.20 ng/g, PFDA of 0.05 ng/g, PFUnA of 0.09 ng/g, PFDoA of 0.06 ng/g, PFOSA of 0.06 ng/g, PFOS of 0.03 ng/g.

^c^Lower confidence interval truncated at 0.

### Human health evaluation

To estimate the potential health risk, doses were calculated from fish tissue concentration based on estimated potential intake and adult body weight. Table 3 summarizes the calculations of exposure dose based on the POC, excluding all PFAS except PFOS concentrations, detected in the fish species, using the 40 g/day ingestion rate established by the current fish advisories established by the Penobscot tribe. The exposure doses were compared to available reference doses to obtain a hazard quotient (HQ). A HQ > 1 indicates levels of the contaminant exceed the reference dose, and a toxicological evaluation may be warranted to determine whether exposure is a health concern and might cause harmful effects [36]. Not all the compounds have an associated reference dose. A total PCB reference dose is not available, so Aroclor 1016 and 1254 were used to evaluate PCBs, with the reference dose for Aroclor 1254 being lower than Aroclor 1016. Both are presented because they represent a range of reference doses with which to compare the total PCBs. The concentrations measured in striped Bass samples exceeded the reference dose for Aroclor 1254, but not Aroclor 1016, resulting in a HQ > 1 for both 2017 and 2018 (Table 3).

**Table 3:** Dose calculations for PCBs, PBDEs, and dioxin/furans (as TEQ), and PFOS.

Species	Year	Total PCBs				BDE 99	Dioxins/Furans/Coplanar PCBs as WHO TEQ 2,3,7,8 – TCDD		PFOS	
		ng/kg BW day ^a^	HQ^b^	HQ^c^	ng/kg BW day	HQ	ng/kg BW day	HQ	ng/kg BW day	HQ^f^
Alewife	2017	7.13	0.1	0.4	0.0976	0.001	0.0135	20^e^	^d^	
	2018	5.15	0.07	0.3	0.0720	0.0007	0.0138	20		
American Shad Roe	2017	3.19	0.05	0.2	0.0218	0.0002	0.0118	20	1.32	13
	2018	4.33	0.06	0.2	0.0271	0.0003	0.00585	8	2.69	27
American Shad Fillet	2017	12.1	0.2	0.6	0.0719	0.0007	0.0130	20		
	2018	7.43	0.1	0.4	0.0774	0.0008	0.0160	20		
Blueback Herring	2017	8.62	0.1	0.4	0.0830	0.0008	0.0112	20	2.09	21
	2018	9.60	0.1	0.5	0.108	0.001	0.0121	20		
Rainbow Smelt	2017	5.12	0.07	0.3	0.0513	0.0005	0.0187	30		
Rainbow Smelt, large	2018	9.28	0.1	0.5	0.0777	0.0008	0.0110	20		
Rainbow Smelt, small	2018	8.44	0.1	0.4	0.0972	0.001	0.114	20		
Striped Bass	2017	40.9	0.6	2	0.0286	0.0003	0.0389	60	1.61	16
	2018	50.1	0.7	3	0.0233	0.0002	0.0352	50	1.13	11
Sea Lamprey	2017	7.37	0.1	0.4	0.0426	0.0004	0.00960	10	3.30	33
	2018	9.04	0.1	0.5	0.0916	0.0009	0.0124	20	2.92	29

^a^Nanograms per kilogram body weight day.

^b^Using RfD for Aroclor 1016.

^c^Using RfD for Aroclor 1254.

^d^Blank space indicates no contaminant detected in the sample.

^e^Shading indicates HQ above 1.

^f^The HQs reflect use of EPA’s RfD from the Office of Water. If the ATSDR intermediate MRL for PFOS was used to derive HQs, HQs ranged from 0.66 to 1.6.

A reference dose for total PBDEs is not available, but the reference dose for BDE-99 was used in the analysis. Several individual congeners have a reference dose, including BDE-153, BDE-47, and BDE-99, all of which were included in the panel of analytes for these fish species. The reference dose for BDE-99 was utilized in the comparison because it has the lowest of the reference doses and all the fish samples had a measurable level in all replicates. All calculations were well below the reference dose, resulting in HQ < 1 for all fish species and indicating minimal potential risk of harmful effects for PBDE at the current ingestion rate.

Dioxin/furans are very toxic compounds [27], so low concentrations can result in potentially hazardous risks. Comparison to the reference dose for 2,3,7,8-TCDD for humans was completed on 2,3,7,8-TCDD TEQs (Table 3). HQs for TEQs of dioxin, furans, and coplanar PCBs exceeded 1 for all species sampled and ranged from 8.4 to 56. Four of the species collected in 2017 had one out of the three replicates with a flagged 2,3,7,8-TCDD result, meaning one of the composite samples was below, at, or near the detection limit. All the samples in 2018 had measurable levels of 2,3,7,8-TCDD, but several other dioxin/furan results were below detectible levels.

PFOS exposure dose calculations are summarized in Table 3. PFOS were used to evaluate potential health risks. Using the current ingestion rate and the EPA’s RfD value, all the samples with measurable levels of PFOS had HQs well above 1, between 11 to 33. A HQ was not derived for PFOS in Alewife, American Shad fillet, or Blueback Herring (2018) because PFOS was not detected in the composite samples. The HQ for PFBA in American Shad roe was well below 1 (not included in Table 3) indicating that consumption of these fish eggs would likely result in minimal harmful risk of adverse effects because of PFBA.

Calculated potential cancer risks are summarized in Table 4 for dioxin and co-planar PCBs. All fish species exceeded the threshold for safe consumption at the moderate cancer risk of 1 × 10^−^^4^. Striped Bass exceeded the higher cancer risk level of 1 × 10^−^^3^ [27]

**Table 4:** Cancer risk estimates for chronic exposure to dioxins/furans/coplanar PCBs as WHO 2,3,7,8 – TCDD TEQ.

Species	TEQ (mg/kg)^a^	Dose (mg/kg BW/day)	Cancer risk^b^
Alewife	2.79 × 10^−5^	1.36 × 10^−8^	6.8 × 10^−4^
American Shad Roe	1.73 × 10^−5^	8.64 × 10^−9^	4.3 × 10^−4^
American Shad Fillet	2.92 × 10^−5^	1.46 × 10^−8^	7.3 × 10^−4^
Blueback Herring	2.33 × 10^−5^	1.17 × 10^−8^	5.9 × 10^−4^
Rainbow Smelt	2.92 × 10^−5^	1.46 × 10^−8^	7.3 × 10^−4^
Striped Bass	7.40 × 10^−5^	3.70 × 10^−8^	18 × 10^−4^
Sea Lamprey	2.20 × 10^−5^	1.10 × 10^−8^	5.5 × 10^−4^

^a^Concentration is average Toxic Equivalents (TEQs) of 2,3,7,8-tetrachlorodibenzodioxin (2,3,7,8-TCDD) using World Health Organization (WHO) toxicity equivalence factors for all samples collected in 2017 and 2018.

^b^Shading indicates a cancer risk above 1 × 10^−4.^

### Impact on wildlife

Converting the fish concentrations to whole fish equivalents allows for the evaluation of potential wildlife impacts from consumption of the tested fish species. American Shad roe are not included in the conversion as the eggs have already been incorporated in the determination of the whole fish values. Table 5 summarizes the results and shows that some wildlife may be adversely impacted by consumption of some of these fish species. The converted whole fish concentration of total PCBs in 2017 and 2018 in Striped Bass exceeded the WV calculated for Mink for Total PCBs of 72 ng/g [11]. A WV for total PBDEs for Kestrel of 8.7 ng/g [11] was exceeded by the 2018 large Rainbow Smelt converted whole fish concentration. The WV for 2,3,7,8 TCDD of 6.5 × 10^−5 ^ng/kg [27] was exceeded by the converted whole fish concentration for all the species measured and represents a potentially harmful source of food to wildlife.

**Table 5:** Converted whole fish concentration of persistent organic chemicals and PFOS in anadromous fish collected from Penobscot river, nanogram per gram [wet weight].

Species	Year	Total PCBs	Total PBDEs	2,3,7,8-TCDD^a,b^	PFOS
Alewife	2017	26.1	2.87	0.161	^c^
	2018	18.8	2.61	0.197	^c^
American Shad Fillet	2017	44.2	4.14	0.180	^c^
	2018	27.2	3.64	0.189	^c^
Blueback Herring	2017	31.6	3.25	0.140	8.91
	2018	35.2	3.45	0.114	^c^
Rainbow Smelt	2017	18.7	3.49	0.256	^c^
Rainbow Smelt, large	2018	34.0	8.89	0.231	^c^
Rainbow Smelt, small	2018	30.9	7.73	0.233	^c^
Striped Bass	2017	150	5.77	0.346	7.50
	2018	183	5.74	0.402	4.81
Sea Lamprey	2017	27.0	1.83	0.160	15.3
	2018	33.1	2.78	0.096	12.4

^a^As each dioxin – like compound has its own conversion factor, a screening level calculation was made using only 2,3,7,8-TCDD.

^b^Shading indicates exceedance of wildlife values.

^c^PFOS perfluorooctanesulfonate; Measured concentrations were below detection limits, so no conversion was calculated.

Table 5 also summarizes the converted whole fish concentrations for PFOS. To evaluate potential risks to wildlife, only PFOS could be investigated as it is the only PFAS with a published WV [32]. The values established by Environment and Climate Change Canada for PFOS were used because no federal US values were available [32]. All of the fish species that contain PFOS, i.e., Blueback Herring, Striped Bass, and Sea Lamprey, exceeded the Canadian WV for mammals of 4.6 ng/g. When compared to the Canadian WV for birds of 8.2 ng/g, Blueback Herring and Sea Lamprey exceeded this value. Based only on PFOS, wildlife may be potentially harmed if consuming these fish.

## Discussion

As a riverine tribal community, the Penobscot Indian Nation members rely on sustenance fishing practices. However, it may be recommended that their unique traditional cultural practices be moderated due to concerns regarding levels of POC including some PFAS contaminants found in the tissue of resident fish species [21]. An earlier study conducted by EPA suggested that the Nation members may be exposed to levels of dioxin/furans and PCBs in resident fish species that may be a health issue if fish were consumed at the traditional intake rate associated with the Nation community [21]. Resident fish species (freshwater fish from the Penobscot River) Chain Pickerel, Yellow and White Perch, Smallmouth Bass, Brown Bullhead, and American Eel contained 0.0037–4.02 pg/g ww of 17 congeners of dioxin/furans as TEQ, and Smallmouth Bass contained 0.432–1.25 ng/g ww of total (142 congeners) PCBs [21]. Consumption of these anadromous fish at or above the amount recommended in the tribal fish advisory may pose cancer and non-cancer risks for the POCs measured.

Dams on the Penobscot River cause disruption of normal fish migrations. Recent restoration efforts, including two dam removals and a constructed bypass from 2012 to 2016, have allowed several anadromous fish species to return to portions of the Penobscot River after a 200 plus year absence [38]. These returning fish could help to reestablish the traditional diet of the Penobscot Indian Nation. Fish advisories assist the Penobscot Indian Nation in reducing the risk from consumption of contaminated fish to tribal members and provide information to assess the sustainability of a traditional Penobscot sustenance diet during current times. Strictly considering the toxicity of the POCs, the current Penobscot Indian Nation fish advisory level of 40 g/day may be inadequate to allow for safe consumption of some of the anadromous fish collected from the Penobscot River [29]. Based on the total PCBs and PBDEs, the current fish advisory is protective for all anadromous fish species except for Striped Bass. However, the current recommended fish advisory might not be protective of health effects from exposure to dioxin-like compounds, including dioxin-like PCBs, and PFOS, via fish consumption [38]. Evaluation of dioxin/furan and PFOS concentrations in fish could be completed again in the future to determine if levels decrease enough to allow the Nation members to return to historical consumption levels. While beyond the scope of this paper, there are benefits to consumption of fish, which some authors have used in risk-benefit evaluation of POCs and fish consumption in tribal and other communities [6, 39].

The fish fillets and portions were analyzed raw [18]. The preparation method may, however, impact the level of contaminants in the ingested fish. It has been found that cooking appears to lower PCBs by 20–30% or as high as 50% if frying [40]. This is not the case for PFAS where cooking does not appear to reduce the amount of contaminant consumed [41]. Taking into consideration the various contaminants tested in the fish, cooking does not appear to be the solution to allow the tribe to consume anadromous fish from the Penobscot River. Over time, contamination may decrease, and further monitoring may be needed to verify safe levels of intake of these fish species.

The anadromous fish had similar levels of PFOS as river and lake fish, as determined in other studies (Lake Trout in Lake Huron [42] Arctic Char in Lake Linnevatnet [43]). Literature was searched for PFAS in fish collected from different systems and concluded that none of the published studies analyzed the same anadromous species. The review indicated that fish samples taken from rivers and lakes had higher concentrations of PFOS than samples taken from the ocean [8, 13, 44–48]. The mean concentrations of PFOS in the anadromous fish analyzed in this study fall in between the results found from rivers, lakes, and ocean fish as reported by others. PFAS levels could be higher in whole fish than in fish fillets [13]. PFOS concentrations in Blueback Herring and Sea Lamprey from 2017, and American Shad Roe and Sea Lamprey from 2018, exceeded the Maine Fish Tissue Action Level of 3.5 ng/g ww [49] which is used by Maine’s Center for Disease Control and Prevention in determining whether to issue a fish advisory. In addition, an ATSDR health consultation evaluated the PFOS exposures and found the average doses calculated in the PFOS samples for American Shad roe, Blueback Herring, Striped Bass and Sea Lamprey exceeded the ATSDR intermediate minimal risk level for PFOS [38, 50].

The health risk to wildlife were also considered in this study. As with human consumption, ingestion of the impacted fish species studied may result in a possible risk to the health of wildlife. Alternative conversion factors [51] for evaluating wildlife impacts have been published which differ from those used for this study; however, a protective approach was taken. The Penobscot Indian Nation is particularly interested in protecting wildlife as they are of cultural significance. Fish advisories do not apply to wildlife, but efforts to reduce contamination of the fish would greatly improve exposure to wildlife and ensure protection of animals that ingest fish from the Penobscot River.

Individually, these contaminants provide varying results with respect to potential risks to the Penobscot Indian Nation community. The data strongly support the need to look at mixtures of chemicals and to consider impacts beyond a single group of compounds, including mercury [18]. To ensure safe consumption of anadromous fish, a cumulative approach (or One Health approach) should be taken which not only considers the needs of humans, but also wildlife. The importance of protecting wildlife often is overshadowed by concentrating on human health. This project demonstrates that wildlife can also be impacted by chemical contamination of fish and their risks can be as great or greater than those of humans. A One Health approach addresses this discrepancy and will lead to a healthier ecosystem.

These data and this analysis have limitations. First, these results are limited to sampling that occurred during two consecutive years. While efforts were made to sample relevant fish that could be consumed, not all fish sizes and species were sampled. Second, this assessment only focused directly on fish consumption, but there could also be other pathways of exposure – for instance, waterfowl or turtles that consume anadromous fish as part of their diet, which are in turn hunted and consumed by people. Third, PFAS health information is being updated rapidly. EPA IRIS assessments for several additional PFAS (PFHxS, PFNA, and PFDA) are currently underway. EPA is also proposing a National Primary Drinking Water Regulation for PFAS including, PFOA and PFOS. As part of this evaluation, EPA is reviewing the peer reviewed science examining noncancer and cancer health effects associated with exposure to these 6 PFAS. Once the PFAS rule has been finalized, the final toxicity assessment, MCLGs, and MCLs may impact One Health assessments of PFAS in the future. While beyond the scope of this paper, ATSDR did evaluate tribal exposure to contaminants in biota and found contaminant levels in turtles and freshwater fish to be of potential health concern at some consumption levels [52].

## Supplementary information

Supplemental Information

## Data Availability

The datasets generated during and/or analyzed during the current study are available from the corresponding author on reasonable request.

## Disclaimer

The views expressed in this article are those of the author[s] and do not necessarily reflect the views or policies of the U.S. Environmental Protection Agency. The United States Environmental Protection Agency, through its Office of Research and Development, funded and managed part of the research described here under EPA Contracts EP-C-15-010 to Pegasus Technical Services, Inc. and EP-C-16-014 to Battelle. Mention of trade names or commercial products does not constitute endorsement or recommendation for use. The findings and conclusions in this report are those of the author(s) and do not necessarily represent the official position of the Agency for Toxic Substances and Disease Registry.
