# Supplementary material for: One Health assessment of persistent organic chemicals and PFAS for consumption of restored anadromous fish
Source: J Expo Sci Environ Epidemiol. 2023 Dec 15;34(6):1035–44. doi: 10.1038/s41370-023-00620-3 (PMC11541783; doi:10.1038/s41370-023-00620-3)
Supplement: Supplementary file 1 — Supplemental Information [file 41370_2023_620_MOESM1_ESM.docx]

**One Health Assessment of Persistent Organic Chemicals and PFAS for Consumption of Restored Anadromous Fish**

**Supplemental Information**

**Moisture Content**

To determine moisture content, a one to five-gram aliquot of homogenized fish tissue was dried at 110±5 degree C using an oven that would maintain this temperature until the final dry weight did not vary by more than ±0.0005 g. The moisture content was determined by subtracting the final weight from the starting weight.

**Lipid Content**

Three grams of fish tissue homogenate were dried with diatomaceous earth for 1 hr and then extracted with 1:1 (v/v) hexane:isopropanol (2 cycles each at 100°C, 1500 psi, 5 min static time, 290 s purge time, and 140% flush volume) using an Accelerated Solvent Extraction (ASE 350) unit (Thermo Scientific Dionex, Waltham, MA). The extract was blown down to a final volume of 25 mL under the flow of nitrogen at 50°C (Zymark TurboVap II, Hopkinton, MA). An aliquot of the extract was air dried in a fume hood, and then placed in a 50°C oven for 90 minutes to complete drying. The lipid weight in the extract was calculated from the final weight subtracted from the starting weight and finally corrected for the mass of tissue extracted. A Laboratory Reagent Blank (LRB) with only hydromatrix and SRM1947 with a lipid content of 10.4 ± 0.5% was also analyzed in each extraction batch. The results were <0.0005% and 10.3 ± 0.9 (n=6), respectively.

**Materials for PCB, PBDE, and Dioxin/furans**

Chemical standards for all target analytes, surrogates and internal standards were purchased from Wellington Laboratories (Wellington Laboratories Inc., ON, Canada). The target analytes included 7 polychlorodibenzo-p-dioxins (PCDDs or dioxins), 10 polychlorodibenzo-p-furans (PCDFs or furans), 27 PBDEs and 32 PCBs. Surrogates included 50 ^13^C_12_ labeled analogue of 6 PCDDs, 9 PCDFs, 8 PBDEs and 27 PCBs congeners. ^13^C_12_ labeled PCB 9, PCB 52, PCB 101, PCB 138, PCB 194, 1,2,3,4-Tetrachlorodibenzo-p-dioxin, and 1,2,3,7,8,9-Hexachlorodibenzo-p-dioxin were used as the internal standard. The accuracy of the standards was verified against other certified sources from Ultra Standard (Elmwood Park, NJ) and AccuStandard (New Haven, CT). The method performance for PCBs and PBDEs detection was validated by analysis of a standard reference material (SRM1947 - Lake Michigan Fish Tissue) and fish tissues fortified with known quantities of all native compounds. Acetone (optima grade), dichloromethane (optima grade), isopropanol (optima grade), hexane (optima grade), sodium sulfate (Certified ACS grade, 10-60 mesh) were purchased from Fisher Scientific (Waltham, MA) while aluminum oxide (basic, Brockmann I, 60A pore size, 70-270 mesh) and silica gel (high purity, 60A pore size, 200-400 mesh) were purchased from Acros Organics (Fair Lawn, NJ) and Sigma-Aldrich (St. Louis, MO), respectively. Granular diatomaceous earth for lipid analysis was purchased from Thermo Scientific Dionex (Waltham, MA).

**Sample Preparation for PCB, PBDE, and Dioxin/furans Analysis**

Accelerated Solvent Extractions (ASE) and cleanup were performed with dichloromethane (DCM) and hexane. Packing materials were rinsed with DCM and baked at varying temperatures/duration according to manufacturer instructions for activation before use for extraction and cleanup steps. Sodium sulfate (baked at 400°C for 4 hr), aluminum oxide (baked at 600°C for 24 hr), and silica gel (baked at 150°C for 4 hr) were stored in a covered flask at 130°C until the time of use (or up to 48 hr) and brought to room temperature just before use.

Adsorbents recommended by EPA Methods 1613, 1614 and 1668, namely silica (10 g, bottom layer) and alumina (30 g, second layer), were layered in a 100 mL ASE cell. The fish tissue homogenate (10 g) was mixed with sodium sulfate (30 g) with a mortar and pestle, dried for 1 hr, and then placed on top of the adsorbent layers (third/top layer). The cells were then spiked with the 50 compound ^13^C_12_ labeled surrogate mix prior to being capped. Samples were extracted with 1:1 (v/v) hexane:DCM (2 cycles each at 100°C, 1500 psi, 5 min static time, 290 s purge time, and 140% flush volume) using a Thermo Scientific Dionex ASE 350 (Waltham, MA). Potential interferences were preferentially adsorbed after passing through the adsorbent layers beneath the fish tissue homogenate during the solvent extraction step and served as the first cleanup step during the extraction process. Target analytes and potential matrix interferences not adsorbed could pass through the adsorbent layers and collect in an ASE bottle. The extracts were reduced to a final volume of 1 mL under the flow of nitrogen at 50°C (Zymark TurboVap II, Hopkinton, MA). A secondary cleanup step was performed by packing a 100 mL ASE cell with alumina (30 g), sodium sulfate (30 g) and 1 mL sample extract from the first ASE step and extracting again using the same extraction conditions as the first ASE step. Interferences, such as lipids present in the extract, were preferentially removed during the second ASE step. The extracts from the second ASE step were also reduced to a final volume of 1 mL under the flow of nitrogen. Finally, alumina cleanup was performed by packing a column with 7 g alumina, pre-eluting with 20 mL DCM which was discarded, pre-eluting with 20 mL hexane which was discarded, adding 1 mL sample extract, eluting with 10 mL hexane, followed by 40 mL 1:1 (v/v) hexane:DCM. After cleanup, the 50 mL extract was blown down to 1 mL and prepared for sample analysis. Ninety microliters of extract were transferred to a 200 µL insert placed into a 2 mL amber GC vial followed by 10 µL of internal standard (IS).

**Instrumentation for PCB, PBDE, and Dioxin/furans Analysis**

PCBs, PBDEs, Dioxin/furans: Method development and sample analysis were conducted using an Agilent 7890A gas chromatograph (GC) (Agilent Technologies, Palo Alto, CA) with a programmable temperature vaporizing (PTV) inlet, a backflush system and CTC PAL autosampler (CTC Analytics AG, Zwingen, Switzerland) coupled to an Agilent 7000B triple quadrupole mass spectrometer with an extractor EI source. Chromatographic separation for most analytes was performed using a capillary DB-5MS UI (20 m x 0.18 mm x 0.18 µm) column (Agilent Technologies, Palo Alto, CA) as the main column. The GC was also equipped with a restrictor column (Agilent CP801510; 1.45 m x 0.15 mm x 0 μm) connected to a backflush system that was capable of reversing flow through the main column and inlet after the final analyte was detected. The source was maintained at 300°C while the two quadrupoles were maintained at 200°C. The GC oven was programmed as follows: 50°C for 1 min, 25°C /min to 200°C for 1 min, 5°C /min to 265°C for 0 min, and 30°C /min to 325°C for 15 minutes (38 minute run time) followed by a 5 minute post run at 325°C where the main column was backflushed with helium at a flow rate of 1.84 mL/min. Helium was used as the carrier gas using the following program: Main column: 1 mL/min for 0.57 minutes, 100 mL/min to 4 mL/min for 2.3 minutes, 100 mL/min to 1 mL/min for 20.3 minutes, and 100 mL/min to 2.5 mL/min until the end of the run; Restrictor column: 4 mL/min for 23.23 min, and 100 mL/min to 6 mL/min until end of run. The PTV inlet was operated in the solvent vent mode for 0.5 minutes to make injections of 25 – 100 μL possible and was programmed as follows: 50°C for 0.01 min, 80°C/min to 70°C for 0.3 min, 720°C/min to 350°C for 2 min, and 25°C/min to 250°C until the end of the run. For octa-, nona- and deca-BDEs, the 20 m column was replaced with a 2 m DB-5ms UI column to reduce high temperature degradation of the target compounds during analysis.

**PCBs, PBDEs, Dioxin/furans Analysis**

The instrument was calibrated using a twelve-point calibration curve (concentration ranging from 0.2 ppt to 125 ppb) prior to each sequence. Isotope dilution mass spectrometry was utilized for the quantification of target analytes by adding a known amount of the labeled compounds to every sample prior to extraction. Optimized mass spectrometric parameters followed approaches previously described.^1-4^ The peak identification of analytes was based on the retention time (±0.05 min) and qualitative to quantitative ion response ratio (±20%). Quality assurance and quality control (QC) during sample analysis were monitored by analyzing a CCV standard solution at regular intervals (every 10 samples). A second source sample prepared from a source of standards different from the source of calibration standards containing a subset of the analytes at known concentrations was also analyzed as a part of each sequence to assure the accuracy of calibration standards and the overall reliability of the analytical process. A lab reagent blank (LRB), lab fortified blank, lab fortified matrix, lab fortified matrix duplicate and SRM1947 were included during sample preparations and analyzed in each batch. The QC samples exhibited acceptable analyte recoveries and repeatability. All target analytes were corrected for blanks using the LRB. Target analytes were corrected for losses during sample processing using the concentrations of the surrogates that were added to the fish samples prior to extraction (i.e., isotope dilution). Target analytes were finally corrected for the mass of the fish used to generate the extracts. Results were reported as ng/g wet weight.

**Materials for PFAS Analysis**

Chemical standards for all target analytes were purchased from Absolute Standards (Absolute Standards, Inc., Hamden CT), labelled surrogates and internal standards were purchased from Wellington Laboratories (Wellington Laboratories Inc., ON, Canada). The target analytes included 13 PFAS compounds of interests, including PFBA, PFPeA, PFHxA, PFHpA, PFOA, PFNA, PFDA, PFUnA, PFDoA, PFOSA, PFBS, PFHxS, and PFOS. Extracted internal standards (surrogates) included labelled analogs for each of the target PFAS compounds, including ^13^C_4_-PFBA, ^13^C_5_-PFPeA, ^13^C_5_-PFHxA, ^13^C_4_-PFHpA, ^13^C_8_-PFOA, ^13^C_9_-PFNA, ^13^C_6_-PFDA, ^13^C_7_-PFUnA, ^13^C_2_-PFDoA, ^13^C_8_-FOSA, ^13^C_3_-PFBS, ^13^C_3_-PFHxS, and ^13^C_8_-PFOS. The internal standards included additional labelled analogs, including ^13^C_3_-PFBA, ^13^C_2_-PFOA, ^13^C_2_-PFDA, and ^13^C_4_-PFOS. The accuracy of the standards was verified against a separate lot number of parent compounds, also purchased from Absolute Standards. Methanol (HPLC grade) was purchased from Fisher Scientific (Waltham, MA) while 7 N NH_3_ in methanol and Envi-Carb cartridges were purchased from Sigma-Aldrich (St. Louis, MO). Farm raised tilapia, used as a clean matrix for procedural blanks and laboratory control samples, was sourced locally (Whole Foods).

**Sample Preparation for PFAS Analysis**

Approximately 5 grams of fish tissue homogenate was weighed into an extraction tube and fortified with labelled extracted internal standards (surrogates). The sample was serially extracted using a Geno/Grinder 2010 (Spex, Metuchen, NJ) with 0.4% NH_3_ in methanol. An aliquot of the combined extract was passed through an Envi-Carb SPE cartridge (Sigma-Aldrich Corp., St. Louis, MO) and eluted with 0.4% NH_3_ in methanol. The extract was concentrated to dryness, fortified with labelled internal standard, and reconstituted to 1 mL with 80:20 methanol/water (V/V). The final extract was further refined with QuEChERS dispersive SPE (Restek Corp., Bellefonte, PA) and transferred for liquid chromatography with tandem mass spectrometry (LC/MS/MS) analysis.

**Instrumentation for PFAS Analysis**

PFAS sample analyses were conducted using a Sciex 6500+ triple quadrupole mass spectrometer (Sciex, Framingham, MA) with a Shimadzu LC system, consisting of dual Nexera X2 LC-20AD pumps, an Nexera X2 SIL-30 AC temperature controlled autosampler, and a Prominence CTO-20AC column oven (Shimadzu Corp., Columbia, MD). Chromatographic separation for all analytes was performed using a Gemini® C18 3 µm; 50 x 2 mm column (Phenomenex, Torrance, CA) as the main column. The LC was also equipped with a delay column ((Phenomenex Luna® C18(2) 5 µm; 30 x 2 mm) connected prior to the injection loop to separate potential background PFAS contaminants. Data were collected in multiple reaction monitoring (MRM) mode monitoring two transitions in negative polarity for all native PFAS except for PFBA and PFPeA. The LC mobile phases consisted of Millipore water with 20 mM ammonium acetate Fisher Scientific (Waltham, MA) as the aqueous phase and methanol as the organic phase. The pumps ramp from 90% aqueous to 1% aqueous and back to 90% aqueous over a programmed ramp with a 7-minute analytical runtime. Table S1 lists the POCs tested.

Table S1: Target POC List

| **Analyte/ Compound** | **Acronym** | **Level of Detection (pg/mL)** | **Range of % Detected**  **2017**  **(all species combined)** | **Range of % Detected**  **2018**  **(all species combined)** |
| --- | --- | --- | --- | --- |
| 2,4'-Dichlorobiphenyl | PCB8 | 11.13 |  |  |
| 2,2',5-Trichlorobiphenyl | PCB18 | 9.67 |  |  |
| 2,4,4'-Trichlorobiphenyl | PCB28 | 17.50 |  |  |
| 3,4',5-Trichlorobiphenyl | PCB39 | 13.59 |  |  |
| 2,2',3,5'-Tetrachlorobiphenyl | PCB44 | 14.77 |  |  |
| 2,2',5,5'-Tetrachlorobiphenyl | PCB52 | 15.43 |  |  |
| 2,3',4,4'-Tetrachlorobiphenyl | PCB66 | 16.61 |  |  |
| 2,3',4,5'-Tetrachlorobiphenyl | PCB68 | 14.54 |  |  |
| 3,3',4,4'-Tetrachlorobiphenyl | PCB77 | 11.94 |  |  |
| 3,3',5,5'-Tetrachlorobiphenyl | PCB80 | 14.54 |  |  |
| 3,4,4',5-Tetrachlorobiphenyl | PCB81 | 13.94 |  |  |
| 2,2',4,5,5'-Pentachlorobiphenyl | PCB101 | 19.59 |  |  |
| 2,3,3',4,4'-Pentachlorobiphenyl | PCB105 | 11.50 |  |  |
| 2,3,4,4',5-Pentachlorobiphenyl | PCB114 | 11.71 |  |  |
| 2,3',4,4',5-Pentachlorobiphenyl | PCB118 | 11.35 |  |  |
| 2',3,4,4',5-Pentachlorobiphenyl | PCB123 | 10.33 |  |  |
| 3,3',4,4',5-Pentachlorobiphenyl | PCB126 | 9.63 |  |  |
| 2,2',3,3',4,4'-Hexachlorobiphenyl | PCB128 | 5.40 |  |  |
| 2,2',3,4,4',5'-Hexachlorobiphenyl | PCB138 | 17.04 |  |  |
| 2,2',4,4',5,5'-Hexachlorobiphenyl | PCB153 | 28.89 |  |  |
| 2,3,3',4,4',5-Hexachlorobiphenyl | PCB156 | 14.94 |  |  |
| 2,3,3',4,4',5'-Hexachlorobiphenyl | PCB157 | 20.95 |  |  |
| 2,3',4,4',5,5'-Hexachlorobiphenyl | PCB167 | 9.93 |  |  |
| 3,3',4,4',5,5'-Hexachlorobiphenyl | PCB169 | 8.32 |  |  |
| 2,2',3,3',4,4',5-Heptachlorobiphenyl | PCB170 | 11.78 |  |  |
| 2,2',3,4,4',5,5'-Heptachlorobiphenyl | PCB180 | 36.73 |  |  |
| 2,2',3,4',5,5',6-Heptachlorobiphenyl | PCB187 | 24.45 |  |  |
| 2,3,3',4,4',5,5'-Heptachlorobiphenyl | PCB189 | 6.38 |  |  |
| 2,3,3',4,5,5',6-Heptachlorobiphenyl | PCB192 | 19.83 |  |  |
| 2,2',3,3',4,4',5,6-Octachlorobiphenyl | PCB195 | 16.56 |  |  |
| 2,2',3,3',4,4',5,5',6-Nonachlorobiphenyl | PCB206 | 28.94 |  |  |
| Decachlorobiphenyl | PCB209 | 15.23 |  |  |
| Total PCBs |  |  | 97-100 | 97-100 |
| 4-Bromodiphenyl ether | BDE3 | 6.79 |  |  |
| 2,4-Dibromodiphenyl ether | BDE7 | 10.75 |  |  |
| 4,4'-Dibromodiphenyl ether | BDE15 | 15.39 |  |  |
| 2,2',4'-Tribromodiphenyl ether | BDE17 | 7.04 |  |  |
| 2,4,4'-Tribromodiphenyl ether | BDE28 | 12.55 |  |  |
| 2,2',4,4'-Tetrabromodiphenyl ether | BDE47 | 20.05 |  |  |
| 2,2',4,5'-Tetrabromodiphenyl ether | BDE49 | 20.95 |  |  |
| 2,3',4,4'-Tetrabromodiphenyl ether | BDE66 | 5.62 |  |  |
| 2,3',4',6-Tetrabromodiphenyl ether | BDE71 | 8.96 |  |  |
| 3,3',4,4'-Tetrabromodiphenyl ether | BDE77 | 8.75 |  |  |
| 2,2',3,4,4'-Pentabromodiphenyl ether | BDE85 | 9.83 |  |  |
| 2,2',4,4',5-Pentabromodiphenyl ether | BDE99 | 13.05 |  |  |
| 2,2',4,4',6-Pentabromodiphenyl ether | BDE100 | 12.96 |  |  |
| 2,3',4,4',6-Pentabromodiphenyl ether | BDE119 | 7.21 |  |  |
| 3,3',4,4',5-Pentabromodiphenyl ether | BDE126 | 10.63 |  |  |
| 2,2',3,4,4',5'-Hexabromodiphenyl ether | BDE138 | 21.60 |  |  |
| 2,2',4,4',5,5'-Hexabromodiphenyl ether | BDE153 | 10.56 |  |  |
| 2,2',4,4',5,6'-Hexabromodiphenyl ether | BDE154 | 19.23 |  |  |
| 2,3,3',4,4',5-Hexabromodiphenyl ether | BDE156 | 18.30 |  |  |
| 2,2',3,4,4',5',6-Heptabromodiphenyl ether | BDE183 | 15.36 |  |  |
| 2,2',3,4,4',6,6'-Heptabromodiphenyl ether | BDE184 | 18.19 |  |  |
| 2,3,3',4,4',5',6-Heptabromodiphenyl ether | BDE191 | 11.72 |  |  |
| 2,2',3,3',4,4',5,6'-Octabromodiphenyl ether | BDE196 | 28.12 |  |  |
| 2,2',3,3',4,4',6,6'-Octabromodiphenyl ether | BDE197 | 28.12 |  |  |
| 2,2',3,3',4,4',5,5',6-Nonabromodiphenyl ether | BDE206 | 83.53 |  |  |
| 2,2',3,3',4,4',5,6,6'-Nonabromodiphenyl ether | BDE207 | 131.05 |  |  |
| Decabromodiphenyl ether 209 | BDE209 | 131.05 |  |  |
| Total PBDEs |  |  | 62-81 | 62-89 |
| 2,3,7,8-Tetrachlorodibenzo-p-dioxin (TCDD) | 2378TCDD | 3.10 |  |  |
| 1,2,3,7,8-Pentachlorodibenzo-p-dioxin (PeCDD) | 12378PeCDD | 6.07 |  |  |
| 1,2,3,4,7,8-Hexachlorodibenzo-p-dioxin (HxCDD) | 123478HxCDD | 10.85 |  |  |
| 1,2,3,6,7,8-Hexachlorodibenzo-p-dioxin (HxCDD) | 123678HxCDD | 7.55 |  |  |
| 1,2,3,7,8,9-Hexachlorodibenzo-p-dioxin (HxCDD) | 123789HxCDD | 8.50 |  |  |
| 1,2,3,4,6,7,8-Heptachlorodibenzo-p-dioxin (HpCDD) | 1234678HpCDD | 12.70 |  |  |
| 1,2,3,4,6,7,8,9-Octachlorodibenzo-p-dioxin (OCDD) | OCDD | 20.36 |  |  |
| 2,3,7,8-Tetrachlorodibenzofuran (TCDF) | 2378TCDF | 4.40 |  |  |
| 1,2,3,7,8-Pentachlorodibenzofuran (PeCDF) | 12378PeCDF | 7.80 |  |  |
| 2,3,4,7,8-Pentachlorodibenzofuran (PeCDF) | 23478PeCDF | 6.82 |  |  |
| 1,2,3,4,7,8-Hexachlorodibenzofuran (HxCDF) | 123478HxCDF | 7.81 |  |  |
| 1,2,3,6,7,8-Hexachlorodibenzofuran (HxCDF) | 123678HxCDF | 8.69 |  |  |
| 1,2,3,7,8,9-Hexachlorodibenzofuran (HxCDF) | 123789HxCDF | 6.38 |  |  |
| 2,3,4,6,7,8-Hexachlorodibenzofuran (HxCDF) | 234678HxCDF | 7.73 |  |  |
| 1,2,3,4,6,7,8-Heptachlorodibenzofuran (HpCDF) | 1234678HpCDF | 8.56 |  |  |
| 1,2,3,4,7,8,9-Heptachlorodibenzofuran (HpCDF) | 1234789HpCDF | 8.42 |  |  |
| 1,2,3,4,5,6,7,8-Octachlorodibenzofuran (OCDF) | OCDF | 21.80 |  |  |
| Dioxin/Furans |  |  | 27-65 | 31-67 |

**PFAS Analysis**

The instrument was calibrated using a seven-point calibration curve (concentration ranging from 100 ng/L to 20,000 ng/L, equivalent to sample concentrations ranging from 0.1 ng/g to 20 ng/g for a 5-gram sample) prior to each sequence. Isotope dilution mass spectrometry was utilized for the quantification of target analytes by adding a known amount of the labeled compounds to every sample prior to extraction. Optimized mass spectrometric parameters followed criteria specified by the Department of Defense (DoD) in the Quality Systems Manual (QSM) version 5.1.1 (2018). The peak identification of analytes was based on the retention time with secondary ion transitions monitored for all native compounds except for PFBA and PFPeA. Quality assurance and quality control during sample analysis were monitored by analyzing a CCV standard solution at regular intervals (every 10 samples). The calibration was verified using a second source sample prepared from a separate standard lot at known concentrations and was also analyzed as a part of each sequence to assure the accuracy of calibration standards and the overall reliability of the analytical process. A procedural blank (PB), lab control sample (LCS), Matrix Spike (MS), and Matrix Spike Duplicate (MSD) were included during sample preparations and analyzed in each batch. Recoveries of extracted internal standards (surrogates) fortified into each sample prior to extraction, were quantified using the internal standards fortified prior to analysis and calculated using known concentrations of surrogates fortified into each sample. The QC samples exhibited acceptable analyte recoveries and repeatability. No target analytes were detected in the PB samples above ½ the limit of quantitation (LOQ). Target analytes were corrected for losses during sample processing using the concentrations of the surrogates that were added to the fish samples prior to extraction (i.e., isotope dilution). Target analytes were finally corrected for the mass of the fish used to generate the extracts. Results were reported as ng/g wet weight. Table S2 lists the PFAS tested.

Table S2: Target PFAS List

| **Analyte** | **Acronym** | **Level of Detection**  **(pg/mL)** | **Range of % Detected**  **(all species combined)** |
| --- | --- | --- | --- |
| Perfluorobutyric acid | PFBA | 0.20 | 0 – 100 |
| Perfluoropentanoic acid | PFPeA | 0.10 | 0 |
| Perfluorohexanoic acid | PFHxA | 0.20 | 0 |
| Perfluoroheptanoic acid | PFHpA | 0.10 | 0 |
| Perfluorooctanoic acid | PFOA | 0.10 | 0 |
| Perfluorononanoic acid | PFNA | 0.20 | 0 |
| Perfluorodecanoic acid | PFDA | 0.20 | 0 – 83 |
| Perfluoroundecanoic acid | PFUnA | 0.20 | 0 – 100 |
| Perfluorododecanoic acid | PFDoA | 0.20 | 0 – 58 |
| Perfluorooctanesulfonamide | PFOSA | 0.20 | 0 – 100 |
| Perfluorobutane sulfonate | PFBS | 0.20 | 0 |
| Perfluorohexane sulfonate | PFHxS | 0.20 | 0 |
| Perfluorooctane sulfonate | PFOS | 0.10 | 0 - 100 |

**Human Health Assessment Equations**

1. D = (C × IR × EF) / BW

Where: D = Exposure Dose (ng/kg/day), C = Contaminant Concentration in fillets or fish portions (ng/g), IR = Ingestion Rate (g/day), EF = Exposure Factor (unitless), BW = Body Weight (kg)

1. HQ = D/RfD

Where: HQ = hazard quotient, D = exposure dose (ng/kg/day), RfD = reference dose (ng/kg/day)

For cancer risks, the toxic equivalencies were calculated using the equation:

1. TEQ = C x TEF

Where: TEQ = toxic equivalent concentration (mg/kg), C = Average Contaminant Concentration in fillets or fish portions (mg/kg), TEF = toxic equivalency factor^5^

Total toxic equivalency was the sum of all TEQs for each dioxin, furan, and dioxin-like PCBs per species.

The exposure dose calculation for dioxin, furan, and dioxin-like compounds was calculated using the TEQ concentrations as specified in the equation:

1. D = TEQ x IR/BW

Where: D = weighted exposure dose (mg/kg/day), TEQ = toxic equivalent concentration (mg/kg), IR = ingestion rate (kg/day), BW = body weight (kg)

Cancer risks were calculated using the equation:

1. Cancer Risk = D x oral slope factor x years of exposure/life expectancy

Where: D = weighted exposure dose (mg/kg/day), oral slope factor = 1.3 x 10^5^ mg/kg/day for TCDD, years of exposure = 30 years, life expectancy = 78 years^6^

**Table S3: 2,3,7,8-TCDD TEQs of Dioxin Like Compounds and PCB Summary**

| **Species** | **Year** | **Dioxins/Furans/Coplanar PCBs, pg/g wet weight as WHO TEQ 2,3,7,8 – TCDD** |
| --- | --- | --- |
| Alewife | 2017 | 0.0269 (0.0239 – 0.03) |
|  | 2018 | 0.0276 (0.0235 – 0.0318) |
| American Shad Roe | 2017 | 0.0236 (0.0152 – 0.032) |
|  | 2018 | 0.0117 (0.0103 – 0.0131) * |
| American Shad Fillet | 2017 | 0.026 (0.0187 – 0.0332) |
|  | 2018 | 0.0319 (0.0307 – 0.0331) |
| Blueback Herring | 2017 | 0.0224 (0.0218 – 0.0231) |
|  | 2018 | 0.0242 (0.0202 – 0.0282) |
| Rainbow Smelt | 2017 | 0.0373 (0.0295 – 0.0452) |
| Rainbow Smelt, large | 2018 | 0.022 (0.0179 – 0.026) |
| Rainbow Smelt, small | 2018 | 0.0227 (0.0203 – 0.0251) |
| Striped Bass | 2017 | 0.0778 (0.0437 – 0.112) |
|  | 2018 | 0.0703 (0.0588 – 0.0818) |
| Sea Lamprey | 2017 | 0.0192 (0.0147 – 0.0238)) |
|  | 2018 | 0.0248 (0.00979 – 0.0398) |

**Statistical Analysis- biplots**

The component axes are the two axis of the plane that provides the most multidimensional separation of the samples. The loading arrows (shown in blue) give an indication of which compounds are correlated with each other, and the position indicates the relative (using ranks) abundance of the compounds. In conjunction with the analysis of similarity performed, there is some indication that year to year samples in the same species had differing fingerprints.

Figure S1: Biplots for PCBs


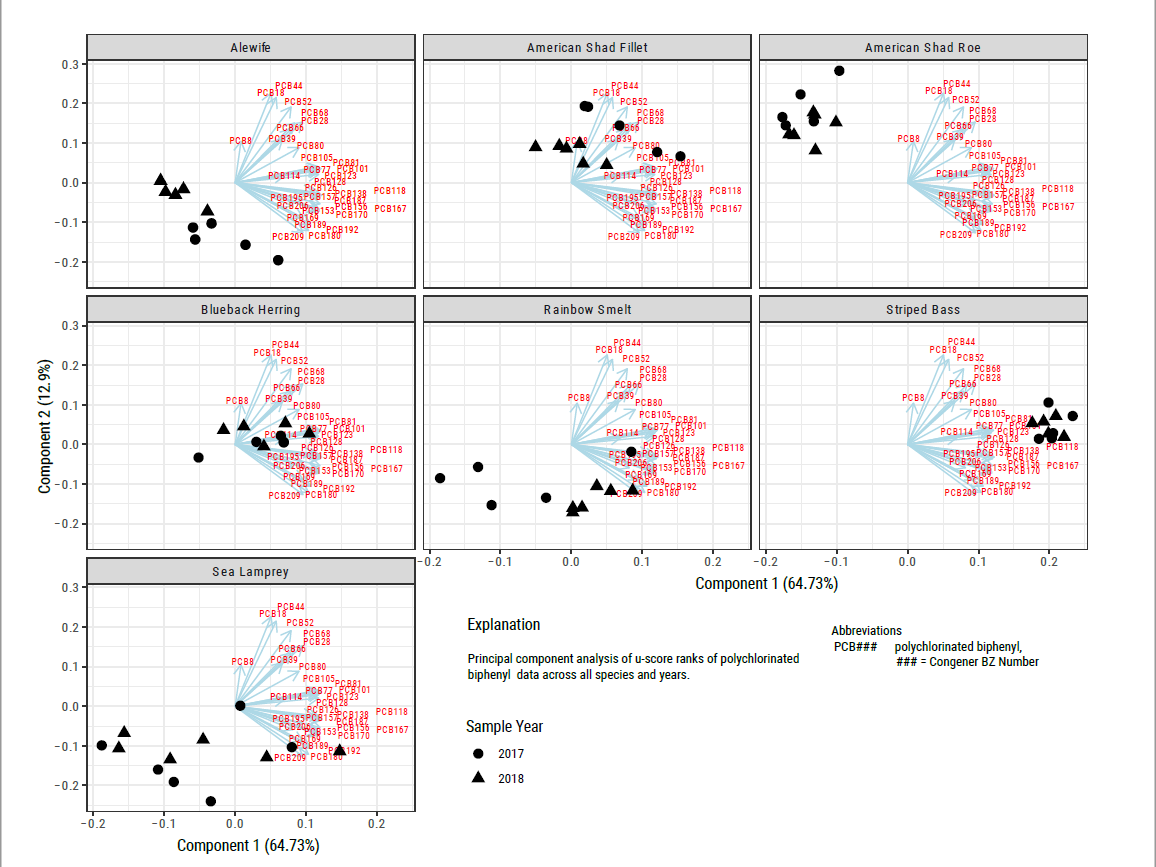


Figure S2: Biplots for PBDEs


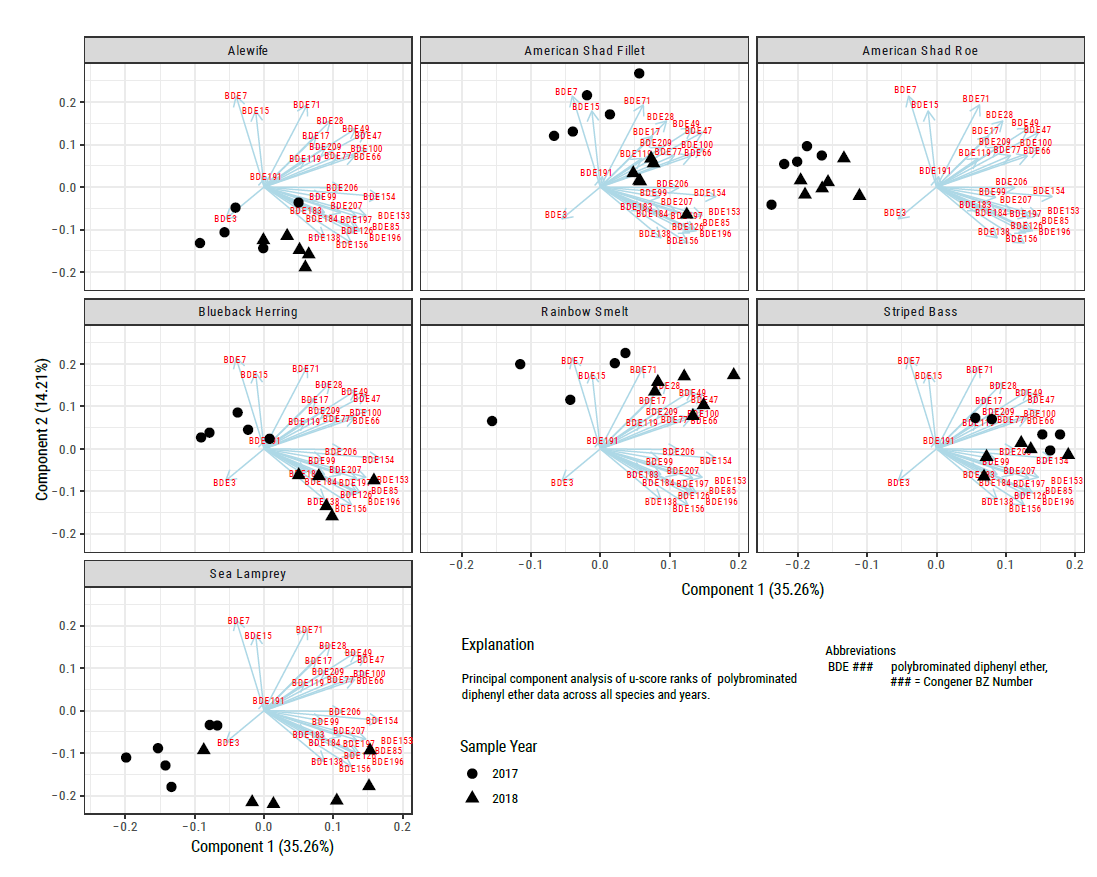


The Striped Bass tend to have higher PCDF’s, and relatively lower PCDDs relative to other samples, since they are along the loadings for the PCDF’s. Whereas the American Shad Roe all tend to be lower in PCDF, but variations are present from relatively high to relatively low in PCDD. The compounds 234678 HxCDF, OCDD, and OCDF were below detectable levels, therefore, not included in the biplot.

Figure S3: Biplots for Dioxin/Furans


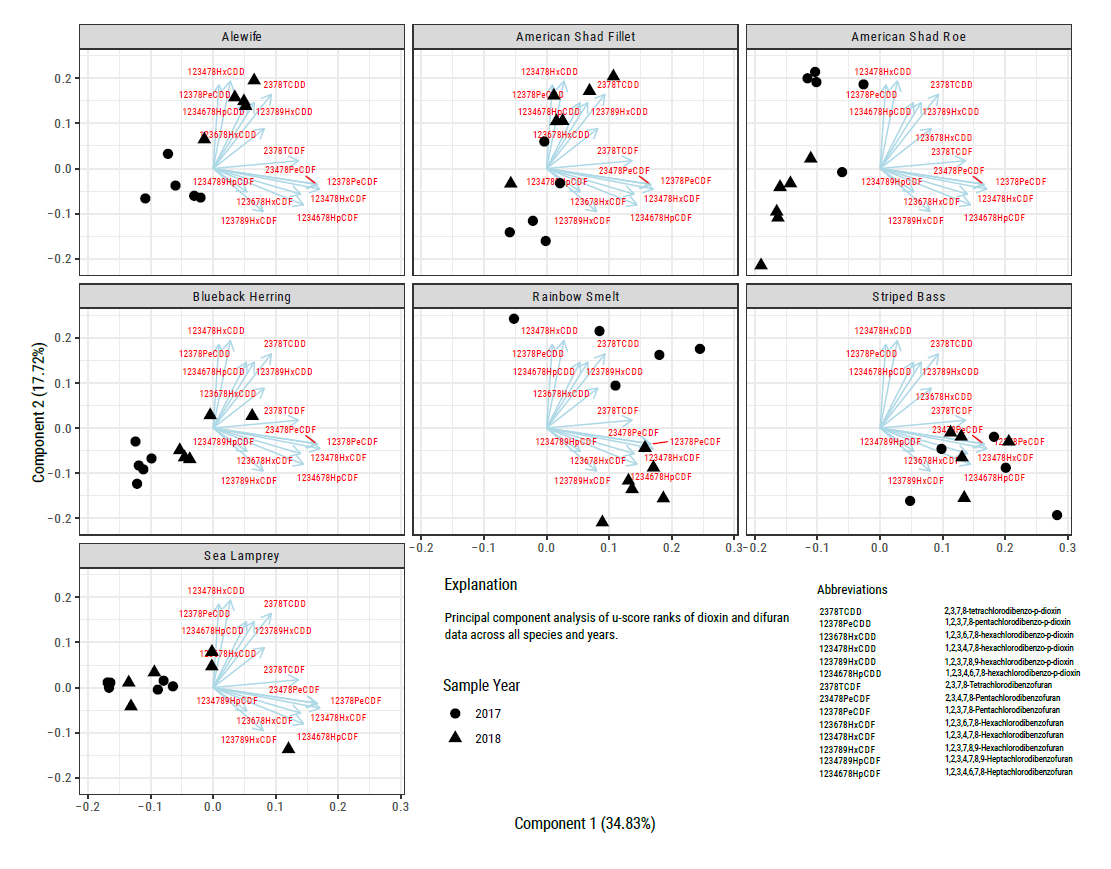


There was variation in PFAS by species and year, with much of the variation in PFBA across species, but Sea Lamprey tended to have the longer chain PFAS compounds.

Figure S4: Biplots for PFAS


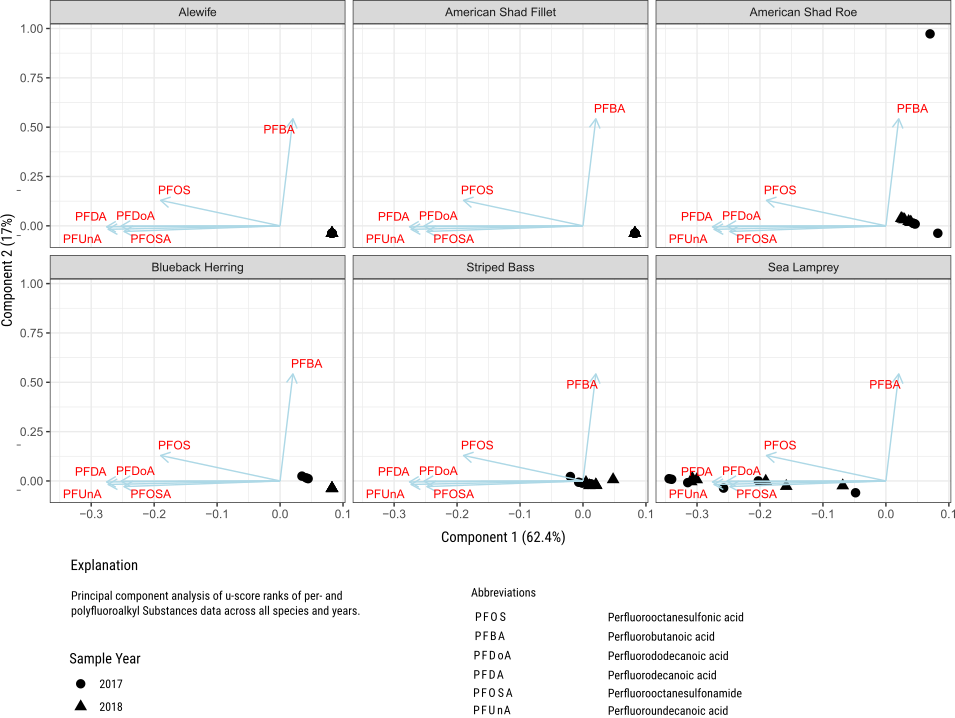


**References**

1. Subedi B, Usenko S. Enhanced pressurized liquid extraction technique capable of analyzing polychlorodibenzo-p-dioxins, polychlorodibenzofurans, and polychlorobiphenyls in fish tissue. Journal of Chromatography A 2012; 1238: 30-37.
2. Riener J. Validation of a Confirmatory GC/MS/MS Method for Dioxins and Dioxin-like PCBS to Meet the Requirements of EU Regulation 709/2014. Agilent Technologies publication 2016: 5991-6590EN.
3. Furst P, Bernsmann T, Baumeister D, Sandy C. Determination of Polychlorinated Dibenzo-p-dioxins (PCDD) and Polychlorinated Dibenzofurans (PCDF) in Foodstuffs and Animal Feed using the Agilent 7000 Triple Quadrupole GC/MS System. Agilent Technologies Publication 2010; 5990-6594EN.
4. Kalachova K, Pulkrabova J, Cajka T, Hajsolva J, Sandy C. Determination of Brominated Flame Retardants (BFRs) in Fish Tissue using an Optimized Extraction/Cleanup Procedure and the Agilent 7000 Triple Quadrupole GC/MS System. Agilent Technologies Publication 2013; 5991-0887EN.
5. U.S. Environmental Protection Agency. Recommended Toxicity Equivalence Factors (TEFs) for Human Health Risk Assessments of 2,3,7,8-Tetrachlorodibenzo-p-dioxin and Dioxin-Like Compounds. EPA/100/R 10/005. Office of the Science Advisor Risk Assessment Forum. [updated 2010 December]. <https://rais.ornl.gov/documents/dioxin_tef.pdf>, last accessed 1/22/21.
6. U. S. Environmental Protection Agency. Exposure Factors Handbook

<https://www.epa.gov/expobox/about-exposure-factors-handbook>, last accessed 5/18/2020.
